# Notch1 activates angiogenic regulator Netrin4 in endothelial cells

**DOI:** 10.1111/jcmm.14240

**Published:** 2019-02-19

**Authors:** Qiang Liu, Thaddeus D. Allen, Wei Song, Youichiro Wada, Corrinne G. Lobe, Ju Liu

**Affiliations:** ^1^ Laboratory of Microvascular Medicine, Medical Research Center Shandong Provincial Qianfoshan Hospital, Shandong University Jinan Shandong China; ^2^ Molecular and Cellular Biology Division, Sunnybrook Health Science Centre University of Toronto Toronto ON Canada; ^3^ Department of Medical Biophysics University of Toronto Toronto ON Canada; ^4^ Tradewind BioScience Daly City California; ^5^ The Research Center for Advanced Science and Technology, Isotope Science Center The University of Tokyo Tokyo Japan

**Keywords:** endothelial cells, Netrin4, Notch1, transcription, transgenic mice

## Abstract

Netrin4 (NTN4) is a chemotropic factor that regulates angiogenesis. We found that endothelial expression of the activated, intracellular domain of Notch1 (NICD1), significantly up‐regulated *NTN4* mRNA as well as intracellular NTN4 protein in both transgenic mice and cultured human umbilical vein endothelial cells (HUVECs). Notch1 activation also increased NTN4 secretion from HUVECs. We subsequently demonstrated that NICD1 bound to CSL (CBF1, Suppressor of Hairless, Lag‐1), a core component of Notch transcription complex, at the −53 element of the human *NTN4* gene promoter. Loss of the −53 element compromised NICD1‐induced *NTN4* expression. Our results suggest a conserved role for Notch signalling in transcriptional regulation of endothelial *NTN4*.

## 1 INTRODUCTION

Netrin4 (NTN4) is a secreted protein that is structurally related to laminins and plays a role in chemotaxis. The Netrin family of proteins contributes either attractive or repulsive guidance cues during neurite outgrowth and neuronal migration.^1^ In addition, NTN4 regulates angiogenesis. *Ntn4* expression is localized to areas of embryonic angiogenesis in zebrafish,^2^ and is functional postnatally during ischaemia‐induced angiogenesis in the rat retina.^3^ Both in vivo and in vitro experiments have demonstrated that stimulation with soluble NTN4 protein markedly promotes angiogenesis by inducing proliferation, migration and tube formation of vascular or lymphatic endothelial cells.^2^, ^4^, ^5^ This is mediated at least in part through activation of the ERK, AKT, JNK and FAK signalling pathways.^2^, ^5^ In mouse models of ischaemia, treatment with Ntn4 protein increases vascular density in ischaemic areas and is beneficial to post‐ischaemic reperfusion.^4^, ^6^ Despite these pro‐angiogenic findings, some studies suggest a contrary role in angiogenesis. Netrin4 suppresses VEGF‐stimulated endothelial cell migration and tube formation by binding to the transmembrane receptor Neogenin (Neo1).^7^ Elevated NTN4 also decreases pancreatic and colorectal cancer growth by inhibiting tumour angiogenesis.^8^, ^9^ In the corneal mouse model, Ntn4 inhibits suture‐mediated neovascularization.^10^ Whether NTN4 acts as a pro‐ or anti‐angiogenic factor may be context dependent. Here, we report that NTN4 is a target of Notch signalling, a conserved pathway which is important in both embryonic and disease‐related angiogenesis.

Notch signalling is highly conserved between species and is critical for differentiation, proliferation and fate determination. Signalling is activated through direct ligand‐receptor interaction and a subsequent cascade of proteolytic events that releases the Notch intracellular domain (NICD). The NICD fragment translocates to the nucleus and triggers target gene transcription by formation of an activation complex with coactivators of the Mastermind family and the DNA‐binding protein CSL (CBF1, Suppressor of Hairless, Lag‐1).^11^ Mammals possess four Notch receptors (Notch1‐4), of which Notch1 and Notch4 are abundantly expressed in endothelial cells. Notch1 is an essential regulator of embryonic vascular development. Both loss and gain‐of‐function studies demonstrate that deregulation of Notch1 leads to severe vascular defects.^12^ Notch signalling co‐ordinates tip and stalk endothelial cell behaviour and is therefore critical for proper interpretation of cues regulating angiogenic guidance and morphogenesis. For example, Notch signalling is necessary for tip cell selection and formation, and increases sensitivity of tip cells to angiogenic factors, such as vascular endothelial growth factor A. Whereas, in stalk cells, Notch activation suppresses the tip cell phenotype and sprouting, and promotes proliferation.^13^ This regulatory effect of Notch in tip and stalk cells ensures the proper formation of vascular networks.

We utilized endothelial‐specific *NICD1* transgenic (Tg) mice and endothelial cell culture models to investigate the transcriptional regulatory effect of Notch signalling on *NTN4*. We found that activation of Notch signalling significantly up‐regulated *NTN4* mRNA and protein expressions, which were observed in both the cell culture and Tg mice. Notch activation also increased the secreted forms of NTN4 in the extracellular environment of cultured endothelial cells. To confirm that *NTN4* is a target of NICD1, we demonstrated that a CSL‐binding element, TGGGAA, at −53 element of the *NTN4* promoter mediated Notch activation of *NTN4*. Our study demonstrates a role for Notch signalling in transcriptional regulation of *NTN4*.

## 2 MATERIALS AND METHODS

### 2.1 Animals

The generation of the endothelial‐specific *NICD1* Tg mice has been previously described.^14^, ^15^ All animal experiments were approved by the Ethics Committee of Shandong Provincial Qianfoshan Hospital.

### 2.2 Cell culture and transduction

Human umbilical vein endothelial cells (HUVECs) were purchased from the ATCC Biosource Center, and cultured in ECM medium (Sciencell, San Diego, CA) with endothelial cell growth supplement (Sciencell), 5% FBS and 1% penicillin‐streptomycin antibiotics at 37°C and 5% CO_2_. Lentiviral particles expressing a gene for the activated *NICD1* or control virus expressing enhanced green fluorescent protein (EGFP), were obtained from GeneChem (Shanghai, China). Lentiviral infection of HUVECs was carried out according to the manufacturer's protocol. HUVECs were screened for puromycin resistance and maintained in culture with 1 μg/mL puromycin (Beyotime, Wuhan, China). Transfection was performed with a lipofectamine 2000 kit (Invitrogen, Carlsbad, CA).

### 2.3 Western blot, Coomassie blue staining and antibodies

Protein samples were prepared in RIPA‐0.1% SDS lysis buffer (Beyotime) and separated on a 10% SDS‐polyacrylamide gel. For Coomassie blue staining, gels were incubated with Coomassie blue staining solution (Beyotime) and washed in distilled water until clear bands appeared. For Western blotting, proteins were transferred onto a polyvinylidene fluoride membrane (EMD Millipore, Burlington, MA). After blocking in 5% skim milk, membranes were incubated with primary antibody at 4°C overnight. The next day, membrane was incubated with HRP‐conjugated secondary antibody (Jackson ImmunoResearch, West Grove, PA). An HRP substrate kit (EMD Millipore) was used to detect chemiluminescense with a fluoroscopic imager (ProteinSimple, San Jose, CA).

### 2.4 Chromatin immunoprecipitation assay

A Chromatin immunoprecipitation (ChIP) assay kit was used to precipitate the target chromatin fragments (EMD Millipore). CSL (Cell Signaling Technology, Danvers, MA) or Notch1 (Abcam, Cambridge, MA) antibody was used for immunoprecipitation with non‐binding isotype control IgG for comparison (Cell Signaling Technology). The immunoprecipitated fragments were amplified with a PCR master mix (Vazyme, Nanjing, China) and analysed on 1% agrose gel.

### 2.5 Dual‐luciferase reporter assay

Human *NTN4* promoter fragments were amplified from human gDNA through polymerase chain reaction (Fermentas, Waltham, MA). The fragments were subcloned into the pGL3‐basic plasmid through Xho I/Hind III digestion. Primer sequences are disclosed in supporting materials (Table S1). Promoter fragments were subcloned into the dual‐luciferase reporter (DLR) reporter plasmid. Human umbilical vein endothelial cells were transfected with the *NTN4*‐promoter DLR plasmid and reference plasmid, pRL‐TK, using a lipofectamine 2000 transfection kit (Invitrogen). Firefly and Renilla luciferase activities were measured with the DLR kit (Promega, Madison, WI).

### 2.6 RNA extraction, cDNA synthesis and quantitative RT‐PCR

Total RNA was isolated using Trizol reagent (Invitrogen) according to the manufacturer instructions. For cDNA synthesis, 1 μg RNA was used in reverse transcription using a synthesis kit (Thermo Fisher Scientific, Waltham, MA). Real‐time PCR was carried out on a Bio‐Rad CFX/96 touch system (Bio‐Rad, Richmond, CA) using SYBR‐Green PCR Master Mix (Tiangen, Beijing, China) with a 20 μL final reaction volume. The relative gene expression was normalized to *β‐actin* and calculated by a comparative Ct method (2^−ΔΔCT^). Primers are listed in the supporting information (Table S1).

### 2.7 Statistical analysis

All quantitative results were calculated from at least three independent experiments. Values represented means ± standard error. Student's *t *test was utilized for statistical analysis.

## 3 RESULTS AND DISCUSSION

### 3.1 Activation of Notch signalling increases NTN4 expression

**Figure 1:**
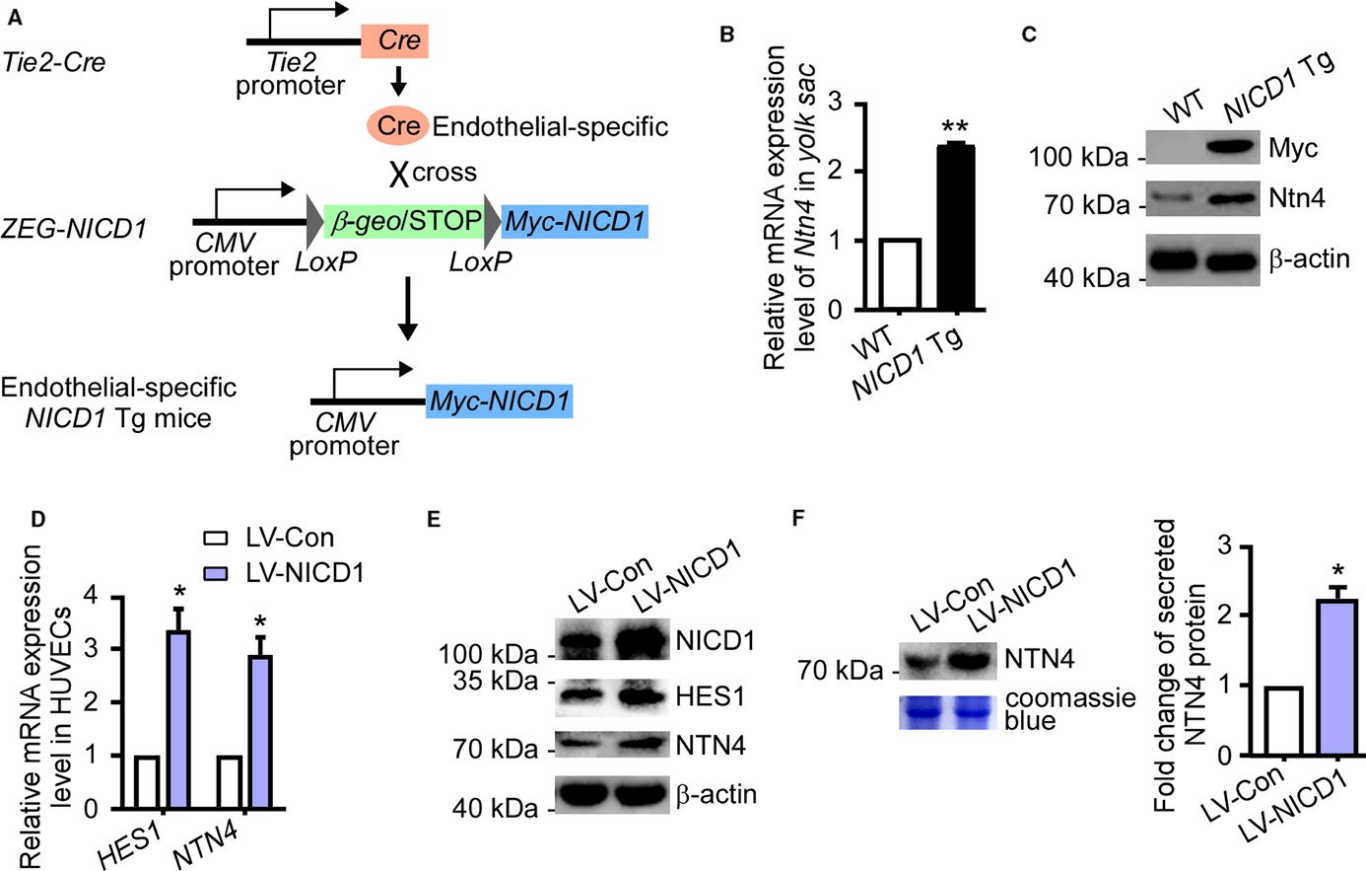
Notch1 activation increase *NTN4 *gene expression. A, Strategy to generate endothelial‐specific *NICD1* transgenic mice. In *ZEG‐NICD1 *Tg mice, the transcription of *NICD1* is initially repressed by a *LoxP* flanked *β‐geo/STOP* signal. To activate expression, the *ZEG‐NICD1* Tg mice have been crossed to *Tie2‐Cre* Tg mice, in which *Cre* transcription is controlled by an endothelial‐specific *Tie2* promoter. In double Tg offspring, the *β‐geo/STOP* signal is removed, leading to endothelial Notch1 intracellular domain (NICD1) overexpression. (B,C) The *yolk sac* was isolated from *NICD1*‐expressing Tg mice (NICD1 Tg) and control littermates (labelled as WT). *Ntn4* mRNA (B) and protein levels (C) were detected by quantitative RT‐PCR and Western blot analysis respectively (β‐actin as loading control). (D‐F) Human umbilical vein endothelial cells (HUVECs) were transduced with NICD1‐expressing lentiviral particles or control virus and puromycin selected to ensure expression. D, mRNA level of *HES1* and *NTN4* detected by qRT‐PCR (data normalized to *β‐actin*). E, Level of cellular NICD1, Netrin4 (NTN4) and HES1 protein in transduced cells. F, Secreted NTN4 protein in culture media detected by Western blot analysis. Coomassie blue staining of the gel was used as loading control. *P < 0.05, **P < 0.01

Both Notch signalling and Netrins regulate cell guidance and angiogenesis. We speculated that there may be a relationship between Notch signalling and the Netrin family protein, NTN4. To test this hypothesis, we first examined the expression of Ntn4 in the *yolk sac* of *NICD1* Tg mice (*NICD1* Tg), in which *NICD1* expression was limited to the zone where its expression was dictated by the endothelial‐specific *Tie2* promoter (Figure 1A). Quantitative RT‐PCR (qRT‐PCR) and Western blot results demonstrated that mRNA (Figure 1B) and protein levels (Figure 1C) of Ntn4 in the *yolk sac* were both markedly elevated in *NICD1* Tg mice. To confirm this regulatory effect of Notch on Ntn4 expression, we activated Notch signalling by infecting HUVECs with lentiviral particles comprising the coding sequence of the Notch1 intracellular domain (NICD1) (Figure S1). We used qRT‐PCR to demonstrate that *HES1*, a canonical downstream target of Notch, was markedly up‐regulated by lentiviral NICD1 (Figure 1D). Concurrently, *NTN4* mRNA expression was significantly elevated, suggesting it is also a downstream target of the Notch pathway (Figure 1D). We confirmed through Western blotting that protein expression was elevated for HES1 and NTN4 in NICD1‐overexpressing endothelial cells. Quantitation of protein elevation in lentivirally transduced HUVECs showed an average of 2.86‐ and 2.52‐fold change for HES1 and NTN4, respectively, compared to control cell lysates (Figure 1E; Figure S2). Because NTN4 is a secreted protein, we asked whether NICD1‐overexpression increases the abundance of secreted NTN4 in the extracellular environment. To confirm this, HUVEC culture media were collected and subjected to Western blot analysis. Netrin4 in NICD1‐overexpressing HUVEC culture was significantly elevated (Figure 1F; Figure S3), indicating that activated Notch1 enhances secretion of NTN4. To examine the regulatory effect of Notch signalling on NTN4 expression under physiological condition, HUVECs were cultured in Delta‐like ligand 4 (DLL4)‐coated dishes. In these cells, Notch signalling was activated, marked by a significant increase of HES1 expression (Figure S4A,B) and NICD1 production (Figure S4B). Transcription of the *NTN4* gene (Figure S4A), cellular (Figure S4B) and secreted NTN4 protein level (Figure S4C) was significantly increased. Moreover, dibenzazepine (DBZ), a specific inhibitor of γ‐secretase and Notch signalling, dramatically reduced the expression of HES1 (Figure S5A,B) and the production of NICD1 (Figure S5B) in HUVECs. Consequently, transcription of the *NTN4* gene (Figure S5A), cellular (Figure S5B) and secreted NTN4 protein level (Figure S5C) was markedly decreased in DBZ‐treated HUVECs. These results imply that NTN4 lies downstream of Notch signalling. We next asked whether NTN4 involves in Notch signalling‐regulated angiogenic activities. The expression of *NTN4* in NICD1‐overexpressing HUVECs was down‐regulated by *NTN4* siRNAs (Figure S6A). As shown in Figure S6B and C, NTN4 knockdown moderately rescues Notch1 activation‐induced reduction of proliferation and migration of HUVECs. The results are consistent with previous studies showing anti‐angiogenic effects of NTN4 in endothelial cells.^7^, ^10^ Together, our study suggests that *NTN4* is a member of Notch1‐targeted gene network in regulation of angiogenesis.

### 3.2 The −53 TGGGAA promoter element is essential for Notch‐induced NTN4 transcription

**Figure 2:**
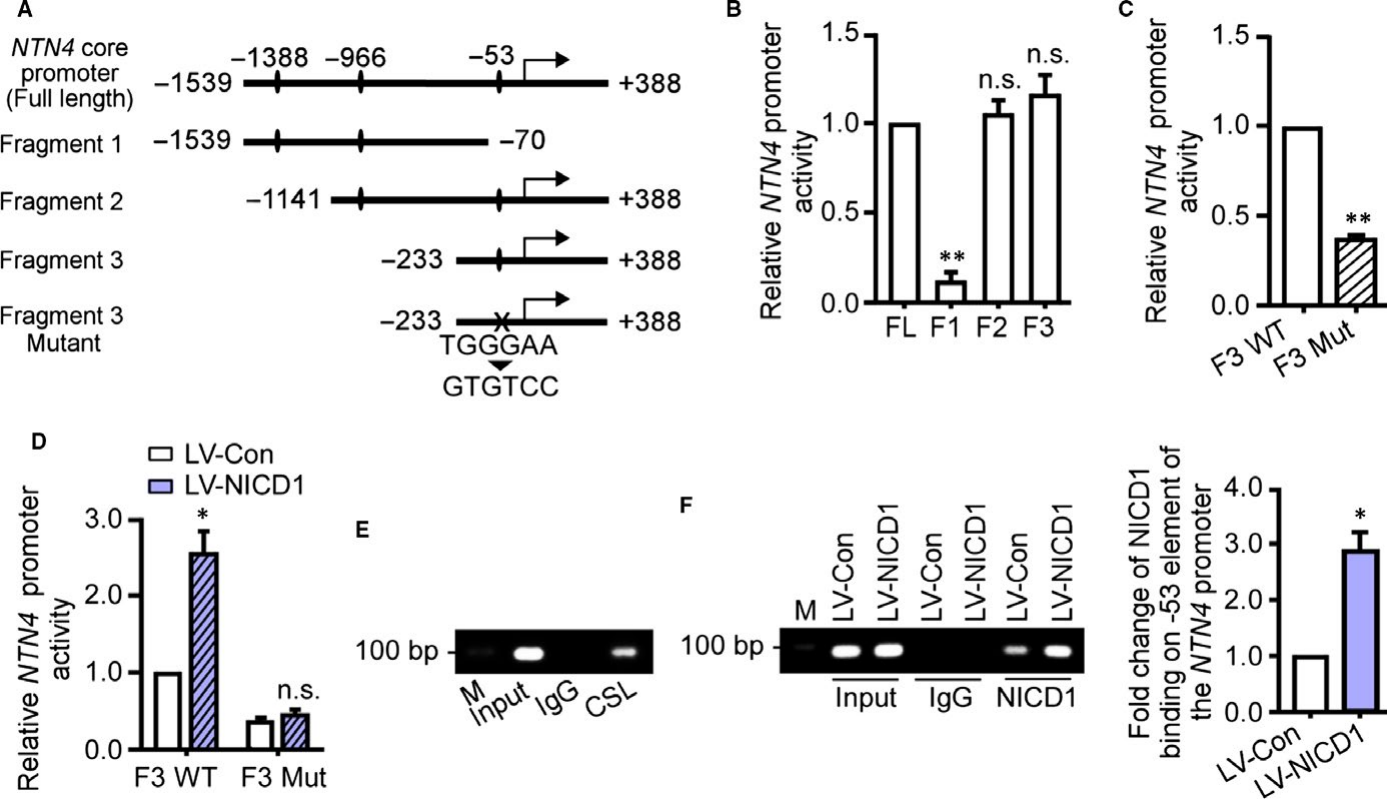
A −53 bp TGGGAA element in the NTN4 promoter is essential for Notch transactivation. A, Human *NTN4* promoter fragments inserted into reporter plasmids for this study. (B,C) Reporter plasmids were transfected into human umbilical vein endothelial cells (HUVECs). Basal activity of corresponding fragments was monitored by dual‐luciferase reporter (DLR) assay. D, The Fragment 3 wildtype and mutant plasmids were cotransfected into HUVECs expressing Notch1 intracellular domain (NICD1). The transcriptional activity was monitored by DLR assay. E, The binding of CSL to the −53 bp element of the *NTN4* promoter was detected by ChIP assay. CSL antibody was used to pulldown CSL. IgG was used as negative control for ChIP. F, The binding of NICD1 protein to the −53 bp element of the *NTN4* promoter was monitored by ChIP assay. The quantitative analysis of band intensity was normalized to the input. *P < 0.05; **P < 0.01; ns, no significance; M, marker. FL, full length

We examined the *NTN4* promoter for sequence that might mediate transactivation downstream of Notch. We found that the CSL‐preferred binding element, TGG/TGAA, occurred at three unique sites in the *NTN4* promoter (Figure 2A) and sought to determine which, if any, functioned to regulate basal activation of the reporter. We constructed a series of DLR assay plasmids that contained at least one of these predicted binding sites (Figure 2A; Figure S7). The DLR assay in HUVECs showed that ablation of the sequence from −233 bp to +388 bp, which contained a predicted CSL site at −53 bp, had a drastic effect on the transcription activation of the *NTN4* promoter plasmid (Figure 2B). Ablation of other fragments failed to alter the reporter activity. To validate that it was the −53 bp CSL‐binding site that played an important role in activating the NTN4 reporter plasmid, we created a mutant that contained five nucleotide substitutions at the −53 binding site (TGGGAA to GTGTCC) (Figure 2A). Deletion of the CSL‐binding site lead to a more than 60% decrease in basal transcriptional activity (Figure 2C).

Next we evaluated the role of Notch signalling in transcriptional activation. As shown in Figure 2D, co‐expression of NICD1 up‐regulated transactivation through the CSL‐binding element at −53 bp (Fragment 3, but not Fragment 3 with TGGGAA to GTGTCC) (Figure 2D). To confirm that NTN4 is a direct Notch target, we studied the binding activity of CSL and NICD1 protein on the predicted −53 bp site by ChIP assay. Either a CSL (Figure 2E) or Notch1 (Figure 2F) antibody was employed for immunoprecipitation. Subsequent PCR amplification of immunoprecipitated chromatin was accomplished with primers that flanked the −53 binding site. Both CSL and Notch1 antibodies enabled identification of a 90 bp band, the size predicted (Figure 2E,F). Moreover, NICD1 overexpression could markedly increase the band intensity, implying that exogenous NICD1 protein was recruited to the −53 bp binding site (Figure 2F). Collectively, these results suggest that *NTN4* is a direct target of a Notch1 transactivating complex. Direct binding occurs at the −53 bp, TGGGAA site of *NTN4 *promoter. This finding provides the first evidence connecting these angiogenic regulatory signals. Notch signalling may work, at least in part, to pattern the vasculature through NTN4.

## CONFLICT OF INTEREST

All the authors declared no conflict of interest.

## AUTHORS’ CONTRIBUTIONS

JL designed the research study; CL, JL contributed essential reagents or tools; QL WS, JL, YW, TA performed the experiments and analysed the data; QL CL, TA, JL wrote the paper. All the authors read and approved the manuscript.
