# Supplementary material for: Notch1 activates angiogenic regulator Netrin4 in endothelial cells
Source: J Cell Mol Med. 2019 Feb 19;23(5):3762–6. doi: 10.1111/jcmm.14240 (PMC6484422; doi:10.1111/jcmm.14240)
Supplement: Supplementary file 1 [file JCMM-23-3762-s001.doc]

**Supporting information**

**Supplementary materials and methods**

*Lentiviral Transduction*

The day before infection, human umbilical vein endothelial cells (HUVECs) were seeded to 12-well platesand grown to an approximately 30% confluency. Culture medium was changed to Opti-MEM (Gibco, Waltham, MA) andplates were seeded with 1×10^6^ TU lentiviral particles. After 12 hours, the Opti-MEM containing lentiviral particles was replaced with DMEM with 10% FBS. Starting at 48 hrs, HUVECs were screened for lentiviral-mediated puromycin resistance with 1 μg/ml puromycin (Beyotime, Wuhan, China) for an additional 7 days.

*Animals*

The *ZEG-NICD1* transgenic mice were previsously established [1]. The *ZEG-NICD1* construct was comprised of a *CMV* promoter, a*loxP*-flanked*β-geo*(*β-galactosidase* and*neomycin*-resistance fusion)/stop signal, the *NICD1* coding sequence, followed by an*IRES* sequence enabling the expression of enhanced green fluorescent protein (EGFP), only after Cre excision. Crosses and screening between *ZEG-NICD1* mice and *Tie2-Cre*micethat lead to the ablation of the*β-geo*/stop signal in F1 generation of double Tg mice and initiate the expression of *NICD1* in endothelial cells, was carried out as previsouly described [2].The *Tie2-Cre*transgenic mice, in which the expression of *Cre* was specifically restricted to endothelial cells by the*Tie2* promoter, were kindly provided by Dr Yanagisawa (University of TexasSouthwestern Medical Center, Dallas, TX) [3].

*Protein Analysis*

Cells or tissues were lysed by sonication in RIPA-0.1% SDS lysis buffer (Beyotime, Wuhan, China) containing 1% protease inhibitor cocktail (MedChemExpress, Shanghai, China). The proteinsamples were denatured by boiling for 10 minutes with loading buffer (Beyotime, Wuhan, China)and dithiothreitol (DTT). Protein samples were run on 10% SDS-polyacrylamide gels.For coomassie blue staining, gels were incubated in coomassie blue staining solution (Beyotime, Wuhan, China) for 10 minutes and washed in distilled water until clear bands appeared. For westerns, protein was transferred to polyvinylidene fluoride (PVDF) membranes (EMD Millipore, Burlington, MA) under a constant 300 mA for 2 hours. PVDF membranes were blocked with 5% skim milkpowder in TBS-0.1% Tween (TBS-T) for 1 hour before incubating with primary antibody at 4 degrees Celcius overnight. The following day, membranes were washed 3 times in TBS-T at room temperature for 15 minutes. They were then incubated with HRP-conjugated secondary antibody for 1 hour at room temperature. After washing three times in TBS-T, an HRP substrate kit (EMD Millipore, Burlington, MA) was used for chemoluminiscent detection on a fluoroscopic imager (ProteinSimple, San Jose, CA). The primary antibodies used were as follows: Notch1 antibody (1:1000; Abcam, Cambridge, MA), NTN4 antibody (for *Homo sapiens*; 1:200; Santa Cruz, Dallas, Texas), Ntn4 antibody (for *Mus musculus*; 1:1000; R&D Systems, Minneapolis, MN), HES1 antibody (1:2000; Abcam, Cambridge, MA); β-actin antibody (ProteinTech, Wuhan, China).

*siRNAs transfection*

*NTN4* and control siRNAs kit were purchased from Genepharma (Shanghai, China). The transfection of siRNAs was performed using lipofectamine 2000 (Invitrogen, Carlsbad, CA), according to the manufecture’s instruction. For 6-well plate, a total amount of 100 pmol siRNAs were transfected into HUVECs.

*Chromatin immunoprecipitation (ChIP) assay*

The ChIP assay kit was purchased from Millipore(Burlington, MA). ChIP assay was carried out following the manufacture’s instructions. Briefly, HUVECs were sonicated 4 times for 10 second-pulses under 15-watt power while on cold ice using a sonicator equipped with a 3-mm tip. CSL (Cell Signaling Technology, Danvers, MA) or Notch1 (Abcam, Cambridge, MA) antibody was applied for immunoprecipitation, and non-specific IgG (Proteintech, Wuhan, China) was used as negative control. The immunoprecipitated fragments were amplified usinga PCR master mix kit (Vazyme, Nanjing, China) on a thermal cycler(Bio-Rad, Richmond, CA) in 20 μl volume. The working parameters were set as: 95 degrees for 3 minutes, 95 degrees for 15 seconds, 60 degrees for 15 seconds, 72 degrees for 30 seconds with 30 PCR cycles. The PCR samples were separated on a 1% acrylamide gel and visualized by a gel imaging system (Tanon, Shanghai, China).

*Plasmid construction*

The human *NTN4* promoter fragments were amplified from human gDNA using a high-fidelity PCR master Mix (Fermentas, Waltham, MA) according to the following steps in 20 μl volume: 98 degrees for 10 seconds, 98 degrees for 5 seconds, 60 degrees for 5 seconds, 72 degrees for 1 minute, all for 35 cycles with a final extension at 72 degrees for 1 minute. Both the PCR samples and pGL3-basic plasmid were digested by Xho I and Hind III (Fermentas, Waltham, MA) and purified by DNA gel extraction kit (Tiangen, Beijing, China). The digested fragments and plasmid were ligated at 22 degrees Celsius using T4 DNA ligase (Fermentas, Waltham, MA). The ligated plasmids were transduced into DH5α and selected on an LB-agar plate containing 100 μg/mL ampicillin (Beyotime, Wuhan, China). The resultant colonies were picked and cultured in LB liquid containing 100 μg/mL ampicillin. Plasmids were isolated by a plasmid extraction kit (Tiangen, Beijing, China) and validated by Xho I-Hind III (Fermentas, Waltham, MA) digestion.

*Dual-luciferase reporter gene assay*

Transfections were carried out on 12-well plates. Transfection of 0.9 μg reporter plasmid and 0.1 μg of the pRL-TK reference plasmid was accomplished with lipofectamine 2000 (Invitrogen, Carlsbad, CA). After 48 hours, HUVECs were collected and lysed with passive lysis buffer (Promega, Madison, WI), and Firefly and Renilla luciferase activity were measured using a dual-luciferase assay kit(Promega, Madison, WI)and a luminometer(Promega, Madison, WI).

*RNA extraction, first-strand cDNA synthesis and quantative Real-time RT-PCR (qRT-PCR)*

Total RNA was isolated using Trizol reagent according to the user manual. For first strand cDNA synthesis, 1 μg of RNA was used for reverse transcription using an RT-PCR kit (Thermo Fisher Scientific, Waltham, MA). Real-time PCR was excuted on a Bio-Rad CFX/96 touch system (Bio-Rad, Richmond, CA) using SYBR green PCR master mix (Vazyme, Nanjing, China) ina 10 μl reaction volume according to the following steps: 95 degrees for 3 minutes, 95 degrees for 15 seconds, 60 degrees for 15 seconds, 72 degrees for 20 seconds, with a total of 40 repeat amplification cycles. Relative gene expression levelswere normalized to *β-actin* and calculated using the comparative *Ct* method (2^-ΔΔCT^).

*MTT assay*

HUVECs were seeded into 96-well flat-bottom plate. Three hours later, 10 μL 5 mg/ml MTT solution (Beyotime, Wuhan, China)was added to each well of the plate and cultured for another 3 hours. The formazan was dissolved with 10% SDS (PH 4.0) overnight. The absorbance was measured at 570 nm in a microplate reader (Thermo Fisher Scientific, Waltham, MA).

*Wound healing assay*

HUVECs were seeded into a 6-well plate 2 days before scratching. A straight scratch was made using a 100 μL pipette tip when the cells reached 100% confluency. The cells were washed once to remove debris and pictures were captured under a microscope. After 12 hours, a new set of pictures were captured at the same position of scratches. The results were analyzed with ImageJ 1.8.0 software (NIH, Bethesda, MD).

**References (Supporting information)**

1. **Liu J, Deutsch U, Jeong J, Lobe CG.** Constitutive notch signaling in adult transgenic mice inhibits bFGF-induced angiogenesis and blocks ovarian follicle development. *Genesis*. 2014; 52: 809-16.

2. **Liu J, Lobe CG.** Cre-conditional expression of constitutively active Notch1 in transgenic mice. *Genesis*. 2007; 45: 259-65.

3. **Kisanuki YY, Hammer RE, Miyazaki J, Williams SC, Richardson JA, Yanagisawa M.** Tie2-Cre transgenic mice: a new model for endothelial cell-lineage analysis *in vivo*. *Developmental biology*. 2001; 230: 230-42.

**Supplementary table**

**Table S1.** Primer list

| **Application** | **Name** | **Sequence (5’-3’)** |
| --- | --- | --- |
| **Plasmid construction** | *NTN4* promoter FL Forward | CCCTCGAGGCAGGATTCGTGCAGACAGTA |
|  | *NTN4* promoter FL Reverse | CCAAGCTTGCAGTTCCATGCGATGCATTT |
|  | *NTN4* promoter F2 Forward | CCCTCGAGTGTGCATCCACCCCTTTGTT |
|  | *NTN4* promoter F3 Forward | CCCTCGAGGCAGCGATTGTTTAGCCGAG |
|  | *NTN4* promoter F1 Reverse | CCAAGCTTGAAACAAGCCCTCCCTCCTC |
|  | *NTN4* promoter F3 Mut Forward | TGGGGGATGGTGTCCGTAGGCGCTA |
|  | *NTN4* promoter F3 Mut Reverse | TAGCGCCTACGGACACCATCCCCCA |
| **ChIP assay** | -53 site Forward | GAGGAGGGAGGGCTTGTTTC |
|  | -53 site Reverse | GCATGCCAAGGGGCTTAAAA |
| **Real-time qPCR** | Human *HES1* Forward | CCTGTCATCCCCGTCTACAC |
|  | Human *HES1* Reverse | CACATGGAGTCCGCCGTAA |
|  | Human *NTN4*Forward | GTACTTTGCGACTAACTGCTCC |
|  | Human *NTN4*Reverse | TCCAGTGCATGGAAAAGGACT |
|  | Human *β-actin*Forward | CACTGTGTTGGCGTACAGGT |
|  | Human *β-actin*Reverse | TCATCACCATTGGCAATGAG |
|  | Mouse *Ntn4*Forward | GCAGGCTTGAATGGAGTAGC |
|  | Mouse *Ntn4*Reverse | GCAGCGTTGCATTTATCACAC |
|  | Mouse *β-actin*Forward | GGCTGTATTCCCCTCCATCG |
|  | Mouse *β-actin*Reverse | CCAGTTGGTAACAATGCCATGT |

**Supplementary figures**

**
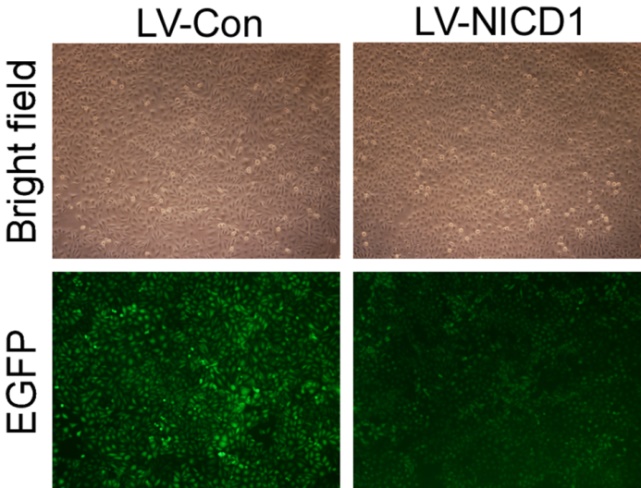
**

**Figure S1.** HUVECs were transduced with NICD1-expressing or control, EGFP expressing lentiviral particles, and visualized under a fluorescent microscope (200×).

**
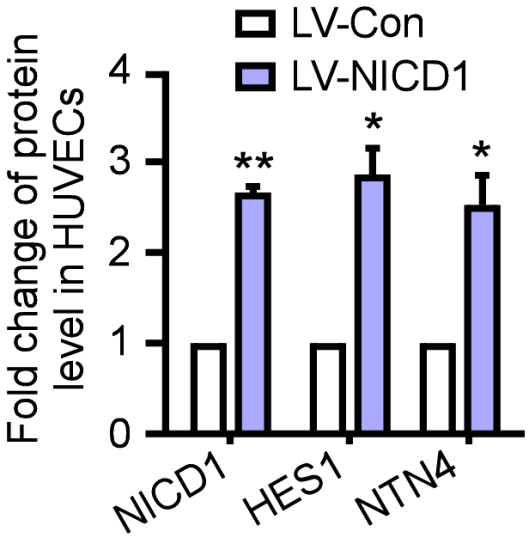
**

**Figure S2.** Quantification of Figure 1E.


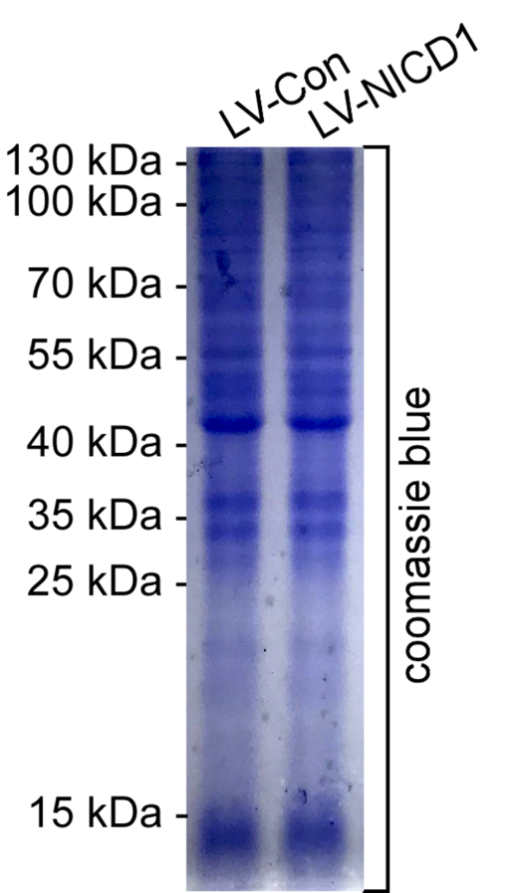


**Figure S3.** Full image of coomassie blue staining of Figure 1F.


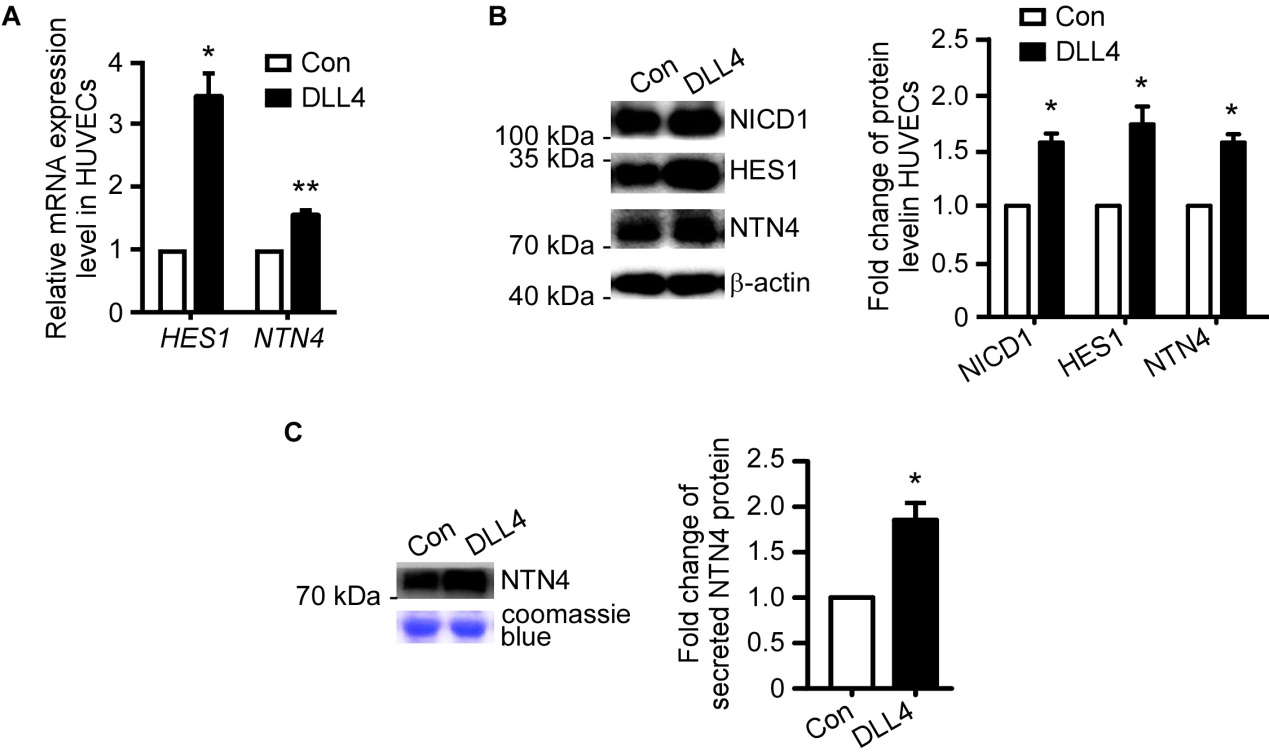


**Figure S4.** DLL4-mediated activation of Notch signaling increases expression and secretion of NTN4. Culture dishes were coated with 5 μg/ml DLL4 (Sinobiological, Beijing, China) in PBS for 12 hours at 4^o^C. HUVECs were seeded into these dishes and cultured for 24 hours. (A) mRNA level of *HES1* and *NTN4* were detected by qRT-PCR (data normalized to *β-actin*). n=4; *, *P*<0.05. **, *P*<0.01. (B) Protein levels of cellular HES1, NICD1 and NTN4 were detected by western blot. β-actin was used as loading control. n=3; *, *P*<0.05. (C) Secreted NTN4 protein in culture media was detected by Western blot. Coomassie blue staining of the gel was used as loading control. n=3; *, *P*<0.05.

**
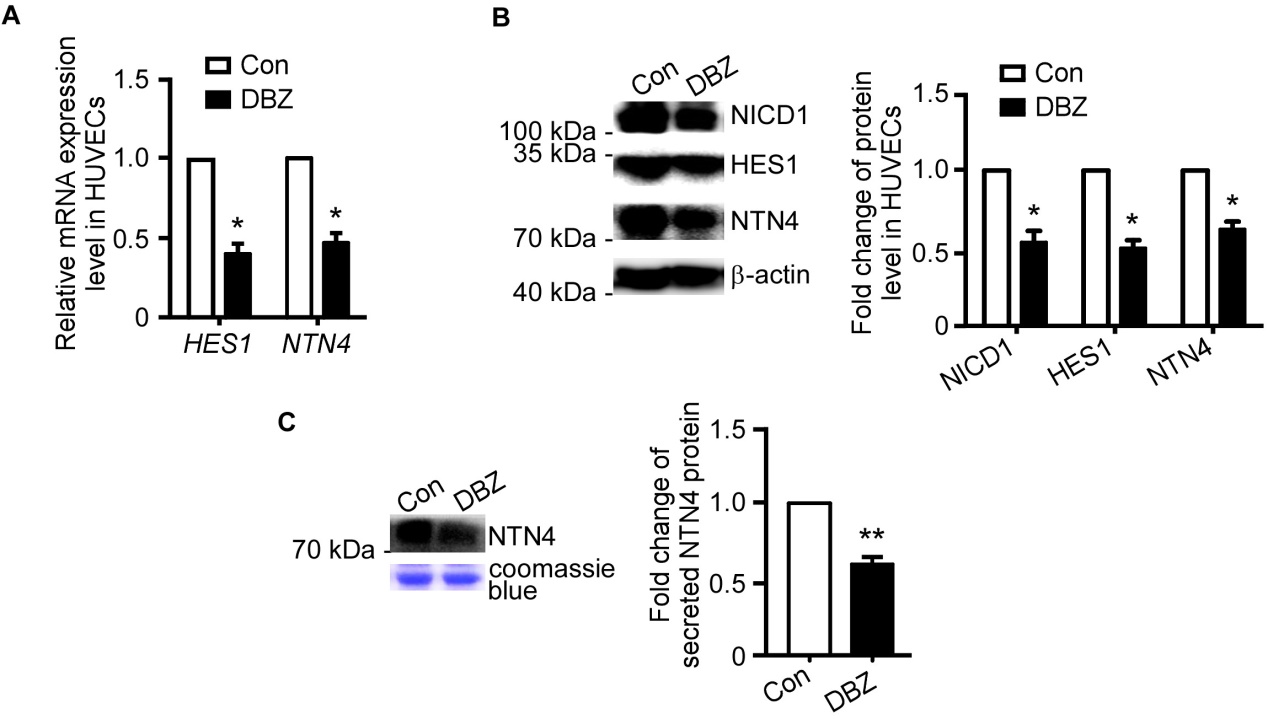
**

**Figure S5.** DBZ treatment decreases expression and secretion of NTN4. HUVECs were treated with 20 μM DBZ for 24 hours. (A) mRNA level of *HES1* and *NTN4* were detected by qRT-PCR. *β-actin* was used as internal control. n=4; *, *P*<0.05. (B) Protein levels of cellular HES1, NICD1 and NTN4 were detected by Western blot (Normalized to β-actin). n=3; *, *P*<0.05. (C) Secreted NTN4 protein was detected by Western blot. Coomassie blue staining of the gel was used as loading control. n=3; **, *P*<0.01.

**
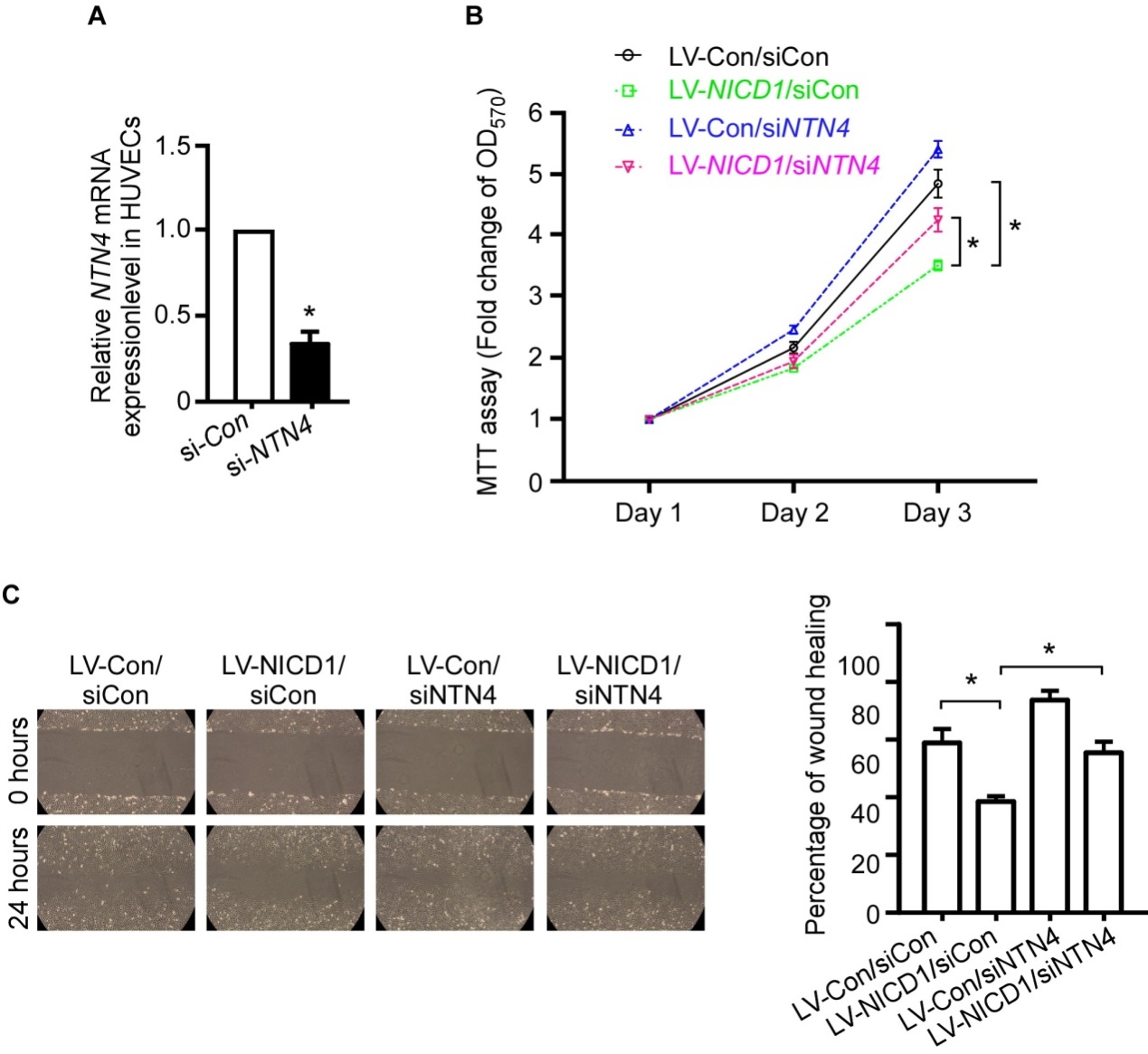
**

**Figure S6.** NTN4 siRNA or control siRNA was transfected into HUVECs with LV-NICD. (A) mRNA level of *NTN4* were detected by qRT-PCR (Normalized to *β-actin*). n=4; *, *P*<0.05. (B) Cell proliferation were evaluated by MTT assay. n=4; *, *P*<0.05. (C) Cell migration was evaluated by wound healing assay. n=4; *, *P*<0.05.


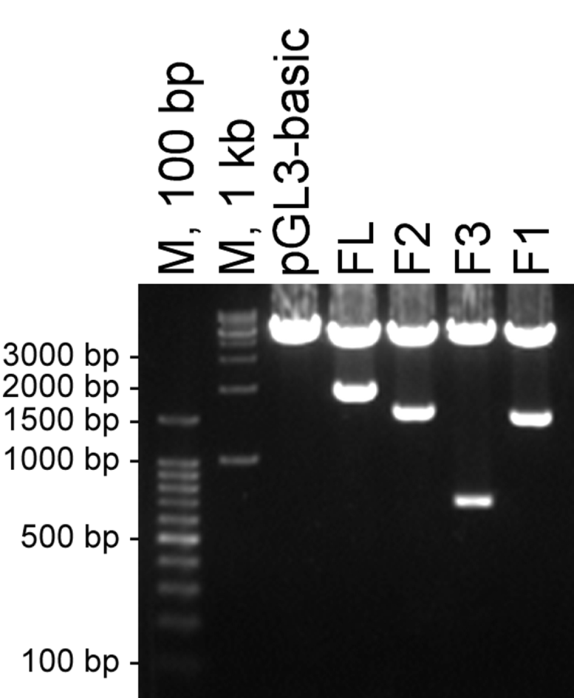


**Figure S7.** Plasmids comprising the full length, different fragments and point-substitutions of *NTN4* promoter were digested with Xho I and Hind III, and analyzed on 1% agrose gel. M, marker. FL, full length. F1, Fragment 1. F2, Fragment 2. F3, Fragment 3.
